# Small RNA Species and microRNA Profiles are Altered in Severe Asthma Nanovesicles from Broncho Alveolar Lavage and Associate with Impaired Lung Function and Inflammation

**DOI:** 10.3390/ncrna5040051

**Published:** 2019-11-02

**Authors:** Ana S. Francisco-Garcia, Eva M. Garrido-Martín, Hitasha Rupani, Laurie C. K. Lau, Rocio T. Martinez-Nunez, Peter H. Howarth, Tilman Sanchez-Elsner

**Affiliations:** 1 Clinical and Experimental Sciences, Sir Henry Wellcome Laboratories, University of Southampton School of Medicine, Southampton General Hospital, Southampton SO16 6YD, UK; anasoraiagarcia@hotmail.com (A.S.F.-G.); emgarrido@ext.cnio.es (E.M.G.-M.); Hitasha.Rupani@porthosp.nhs.uk (H.R.); L.C.Lau@soton.ac.uk (L.C.K.L.); rocio.martinez_nunez@kcl.ac.uk (R.T.M.-N.); 2 NIHR Southampton Respiratory Biomedical Research Unit, Southampton Centre for Biomedical Research MP812, Southampton General Hospital, Southampton SO16 6YD, UK

**Keywords:** microRNAs, severe asthma, exosomes, lung function, FEV_1_, eosinophils, neutrophils

## Abstract

MicroRNAs are known to regulate important pathways in asthma pathology including the IL-6 and IFN pathways. MicroRNAs have been found not only within cells but also within extracellular vesicles such as exosomes. In this study, we particularly focused on microRNA cargo of nanovesicles in bronchoalveolar lavage of severe asthmatic patients. We extracted nanovesicle RNA using a serial filtration method. RNA content was analyzed with small RNA sequencing and mapped to pathways affected using WebGestalt 2017 Software. We report that severe asthma patients have deficient loading of microRNAs into their airway luminal nanovesicles and an altered profile of small RNA nanovesicle content (i.e., ribosomal RNA and broken transcripts, etc.). This decrease in microRNA cargo is predicted to increase the expression of genes by promoting inflammation and remodeling. Consistently, a network of microRNAs was associated with decreased FEV_1_ and increased eosinophilic and neutrophilic inflammation in severe asthma. MicroRNAs in airway nanovesicles may, thus, be valid biomarkers to define abnormal biological disease processes in severe asthma and monitor the impact of interventional therapies.

## 1. Introduction

Exosomes are extracellular membrane-derived vesicles, of approximately 30–150 nm in diameter, which are generated and released in a tightly regulated manner [1]. They may contain DNA, proteins, lipids, and RNA molecules, including microRNAs, and participate in intercellular communication. MicroRNAs are short (~22 nucleotides) non-coding single stranded RNAs that regulate gene expression by targeting mRNAs and blocking their translation or inducing their degradation [2]. We have described that microRNAs can affect important pathways (IL-6 and Interferon production) in airway cells from asthmatic patients [3,4]. Over 750 entries for miRNAs have been described in airway exosomes, which highlighted their potential importance in regulating airway cell function. Exosomal microRNAs have been evaluated in exhaled breath, bronchoalveolar lavage, and sputum supernatant samples [5,6,7]. The exosomal microRNA cargo in bronchoalveolar lavage fluid (BALF) is dysregulated in mild asthma [6]. However, no assessments have been undertaken of BALF exosomal microRNA profiles in severe asthma, which is a disease that is only partially responsive to treatment and is recognized to be associated with epithelial dysfunction and altered luminal inflammation [8].

## 2. Methods

### 2.1. Subjects and Sampling

Study volunteers were all non-smokers and were either healthy volunteer subjects or severe asthma patients under clinical care, for whom other causes of persistent symptoms had been excluded. All bronchoscopies were done in the morning. Severe asthmatic patients were on high dose inhaled steroids and additional controller therapy, which was normally a LABA. The patients took varying proportions of LAMA and LTRA with some also being on oral steroids. Severe asthmatic samples were collected in a period of clinical stability. Patient demographics are shown in Table 1. Healthy volunteers (specifically recruited for research purposes) all had normal bronchial reactivity and no history of present or past asthma symptoms as well as no other respiratory conditions.

BAL fluid was filtered using a 100-µm nylon filter and then centrifuged at 400 rcf for 10 min at 4 °C. The supernatant was removed, aliquoted, and stored at −80 °C. The cells were resuspended in PBS and counted using a Neubauer hemocytometer. An aliquot of cells was used to prepare centrifuge slides.

### 2.2. Ethics

Ethical approval was given by the Southampton and South Hampshire Research Ethics Committee (ethics numbers 05/Q1702/165 and 08/H0502/6). All subjects gave written informed consent.

### 2.3. Nanovesicle Small RNA-Sequencing

#### 2.3.1. Extraction of Nanovesicle RNA from BAL

Nanovesicle RNA was extracted from twenty millilitres of bronchoalveolar lavage using the ExoMir kit (Biooscientific, Austin, TX, USA) following manufacturer’s instructions. BAL was loaded into a 20-mL syringe and passed through a sequence of two filters. The pore size of the top filter was 0.2 μm whereas the bottom filter’s pore size was 0.02 µm. RNA from each of the filters was harvested separately using 1 ml of Bioopure reagent. RNA extraction was then performed according to the manufacturer’s instructions. Extracts from the bottom filter were used for small RNA-sequencing. 

#### 2.3.2. Small RNA-Sequencing

Total RNA samples from human BAL fluid nanovesicles were submitted to Ocean Ridge Biosciences (Deerfield Beach, FL, USA). RNA was quantified using Ribogreen and RNA quality was determined employing a Bioanalyzer assay on an Agilent RNA 6000 Pico microfluidic chip. All samples were digested with RNase-free DNase I, and re-purified on RNeasy MinElute columns (Qiagen, Hilden, Germany) using an alternative high-ethanol binding condition (to preserve low molecular weight RNAs).

Template DNA molecules suitable for cluster generation were prepared from the re-purified RNA samples using the NEBNext Small RNA-Seq Library Preparation Kit (New England BioLabs, Ipswich, MA, USA) according to the manufacturer’s instructions, with an additional step following the RNA linker ligation after cDNA synthesis. cDNA fragments were fractionated using a 12% acrylamide-urea gel and 58–88 nt fragments, corresponding to inserts of 11–41 nt, were excised and purified to ensure a minimum size range of 17–35 nt inserts was recovered for each sample. Libraries were electrophoresed through a freshly cast 6% native polyacrylamide gel and the library fragments of an appropriate size (~130–160 nt) were purified and recovered by agitation of the fragments overnight at 37 °C at 200 rpm in elution buffer, passage of the eluate through a 0.45-μm filter, and ethanol precipitation. The quality and size distribution of the amplified libraries were determined utilizing an Agilent High Sensitivity DNA Bioanalyzer microfluidic chip. Libraries were quantified using the KAPA Library Quantification Kit (KK4824, Kapa Biosystems, Boston, MA, USA). 

Libraries were pooled at equimolar concentrations and diluted prior to loading onto the flow cell of the cBot cluster station (Illumina Inc., San Diego, CA, USA). The libraries were extended and bridge amplified to create single sequence clusters using the TruSeq SR Cluster Kit version 3—HS (Illumina Inc., San Diego, CA, USA). The flow cell carrying amplified clusters was loaded on the HiSeq 2000 sequencing system (Illumina Inc., San Diego, CA, USA) and sequenced with 50 bp single-end reads using the TruSeq SBS Kit version 3—HS (Illumina Inc., San Diego, CA, USA). In addition, 10% ΦX174 phage DNA was spiked in all sequencing lanes for sequencer calibration. Real time image analysis and base calling were performed on the instrument using the HiSeq Sequencing Control Software (HCS). CASAVA software version 1.9 was used for demultiplexing and producing FASTQ sequence files. 

Raw FASTQ sequence files generated using CASAVA version 1.9 software were transferred to a dedicated Linux-based computing cluster for further processing. Low quality sequences were filtered out. FASTX application [9] was used to trim adapter sequences from the 3′ end of the sequence read, discard any sequence of less than 17 nucleotides after trimming, and collapse identical reads into single entries while retaining the read count for each unique sequence. Non-redundant sequences were then aligned to genomic and mRNA sequence using bowtie 2 [10] sequences with perfect-match and 1 nt mismatch alignments were retained for further analysis. The mapped sequences were aligned to common and abundant non-coding RNAs (tRNAs, rRNAs, and snoRNAs), again using bowtie 2. The genome-mapped sequences were further aligned to both mature microRNA and primary microRNA databases (miRBase 20.0), using OMAP# alignment software developed at ORB.

Alignment result files were parsed by a custom Perl script to generate FASTA files containing the read count for each unique sequence and information about the target from the database (e.g., microRNA name and ID). The FASTA files were tabulated using an additional custom OMAP Perl script in order to determine the raw sequence read count for each target database entry. The raw reads were converted to reads per million mapped reads (RPM). The RPM values were filtered to retain a list of small RNAs with a minimum of 10 mapped reads (detection threshold) in 25% of samples. Missing values were replaced with the average RPM value equivalent to one read. 

Sequencing data are uploaded onto GEO and available upon request. 

### 2.4. Pathway Analysis for Differentially Expressed microRNAs in Severe Asthmatic Nanovesicles

Pathway analysis for microRNAs associated with pre-bronchodilator FEV_1_, eosinophilic, or neutrophilic infiltration was preformed using miRNA Target Filter analysis of Ingenuity Pathway Analysis (IPA, Qiagen). Five miRNAs correlated with FEV_1_ and were mapped to 2136 target genes, three miRNAs correlated with neutrophilic inflammation and were mapped to 956 target genes, and three miRNAs correlated with eosinophilic inflammation and were mapped to 1409 target genes. Then, pathway analysis was performed using IPA and altered pathways predicted with a raw *p* value of less than 0.010 were selected.

Further pathway analysis was performed by Ocean Ridge Biosciences, USA. Selected microRNAs with a potential difference between the healthy vs. SA group based on a raw *p*-value of less than 0.01 were selected for analysis. 

Separate queries of TargetScan version 6.2 and mirDB version 4.0 (Refs for TS and miRDB) were performed. Maximum prediction score for each microRNA-gene pair from each database was reported. Unique microRNA-gene target combinations shared between TargetScan and mirDB for each original microRNA list were selected.

Tables showing the list of microRNA-gene combinations expanded to include one row for each unique microRNA binding site, which were prepared, and additional details of binding sites were reported from TargetScan.

Unique gene lists from the steps above, for each original microRNA list, were reported and gene set enrichment analysis using WebGestalt software [11] was performed.

### 2.5. Western Blotting

The aqueous phase of nanovesicle lysates resulting from chloroform extraction was removed for RNA analysis. The phenol phase was used for protein extraction. Protein was subjected to PAGE. Antibodies employed were anti-CD63 and anti-calnexin (Santa Cruz Biotechnology, Dallas, TX, USA) and anti-rabbit HRP conjugated (Dako-Agilent, Santa Clara, CA, USA). 

### 2.6. Validation of Small-RNA Sequencing Using qPCR

Reverse transcription of total RNA and qPCR on the resulting cDNA, was performed using the Taqman microRNA individual assays following manufacturer’s instructions (ThermoFisher 4427975, Waltham, MA, USA, for hsa-miR-155 and 4427975 for hsa-miR-27a). Additionally, 1.5 µL RT microRNA specific primers, 0.30 µL of dNTPs (100 nM), 1 µL of multi-scribe reverse transcriptas (50 U/µL), 0.75 µL of 10X RT buffer, 0.2 µL of RNase inhibitor (20 U/µL), and 1 µL of nuclease-free water, per sample, were mixed and 4.5 µL of this mix was combined with 2 µL of 5 ng/µL total RNA. Samples were loaded onto a DNA Engine Tetrad 2 PTC-0240 Peltier Thermal Cycler (Bio-Rad, Hercules, CA, USA) and run for 30 min at 16 °C, 30 min at 42 °C, and 5 min at 85 °C. To prepare the RT-qPCR mastermix, 0.25 µL of 20X TaqMan microRNA assays qPCR primers, 0.5 µL of undiluted RT product, 2.5 µL of TaqMan Universal Master Mix II, No AmpErase UNG (2X), and 1.75 µL of nuclease-free water per sample were mixed. In addition, 5 µL of this reaction was added to each well of a 384-well plate. The plate was sealed with MicroAmp Optical Adhesive Film. Plate was run on Applied Biosystems 7900 HT Fast Real-Time (Applied Biosystems, Foster City, CA, USA) for 10 min at 95 °C and then 40 cycles of 95 °C for 15 s and 60 °C for 15 s.

## 3. Statistics

### 3.1. Statistical Analysis

Fold changes between healthy subjects and severe asthmatics nanovesicle microRNA levels were calculated by comparing the mean values from the two groups. Significance was calculated using 1-Way ANOVA. FDR and *p* values were obtained using NIA Array analysis software. A significance was established at *p* ≤ 0.05.

To assess statistical relationships between variables, the Spearman’s rank correlation coefficient was calculated. A correlation was deemed significant when *p* ≤ 0.05. Statistical analysis was performed using GraphPad Prism 6 (GraphPad Software, Inc, San Diego, CA, USA).

### 3.2. Power Calculation

Power of the study was determined based on microRNAs significantly correlating with FEV_1_, neutrophil, and eosinophil infiltration. Effect size for these microRNAs varied between 0.45 and 0.99 with α error probability set at 0.1. Calculations performed using G*Power 3.1.9.4 software (Universitat Dusseldorf) [12].

## 4. Results

### 4.1. Small RNA Cargo Is Altered in Severe Asthma Nanovesicles

**Figure 1:**
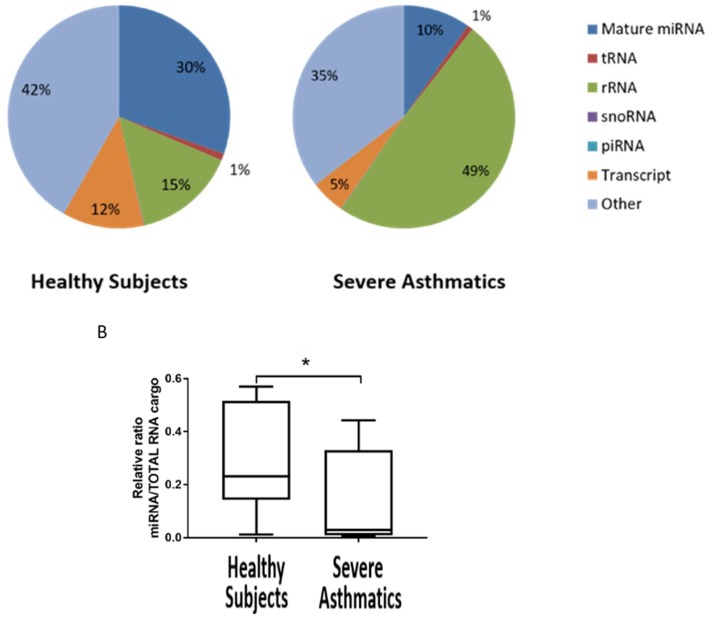
Small RNA species other than microRNA are increased in SA nanovesicles. (**A**). Proportion of RNA species present in BALF nanovesicles of SA and HC. (**B**). Ratio of microRNA cargo in nanovesicles compared to total RNA cargo (* *p* < 0.05). Statistics (*p* value and FDR) according to NIA Array analysis software (*p* ≤ 0.05).

We purified nanovesicles from SA patients (*n* = 12) and healthy subjects (*n* = 8) and extracted RNA. Nanovesicles were characterised by RT-qPCR (determining presence of microRNAs miR-155 and miR-27a and low expression/absence of RNU44 and 18S) and WB (absence of Calnexin and presence of CD63) (Supplementary Figure S1). MiR-155 and miR-27a were used as positive controls of the presence of microRNAs since they are relatively abundant in airway cells [3,4]. Confirming our RT-qPCR data, analysis of quality RNA by the Bioanalyser showed an absence of large rRNA and a peak of small RNA in the region of 25 to 100 nt, as expected (Supplementary Figure S2). We wondered whether nanovesicles from SA are being loaded with different species of small RNAs. We took advantage of the fact that small RNA-seq data allows for this comparison since it detects not only microRNAs but also other species of small RNAs such as tRNA, rRNA, piRNA, snoRNA, and pieces of mRNA transcripts. Strikingly, our analysis showed that mature microRNA content decreases from 30% in HC to 10% in SA while small ribosomal RNA (rRNA) increases from 15% in HC to 49% in SA (Figure 1A). This increase in small RNA cargo of nanovesicles in SA could be explained by an increase in small fragmented rRNA, since no 18S or 28S ribosomal RNA peaks were evident in the Bioanalyzer traces (Supplementary Figure S2). We then compared the ratio of microRNA/total small RNAs in each group and showed a statistical difference indicating that SA nanovesicles contain a lower ratio of microRNAs in their nanovesicles (Figure 1B, *p* < 0.0287). This lower ratio may be the cause of the observed reduced cargo of microRNAs and could have functional consequences for the pathology of asthma.

### 4.2. Reduced Load of microRNAs in Nanovesicles from Severe Asthmatics Patients

**Figure 2:**
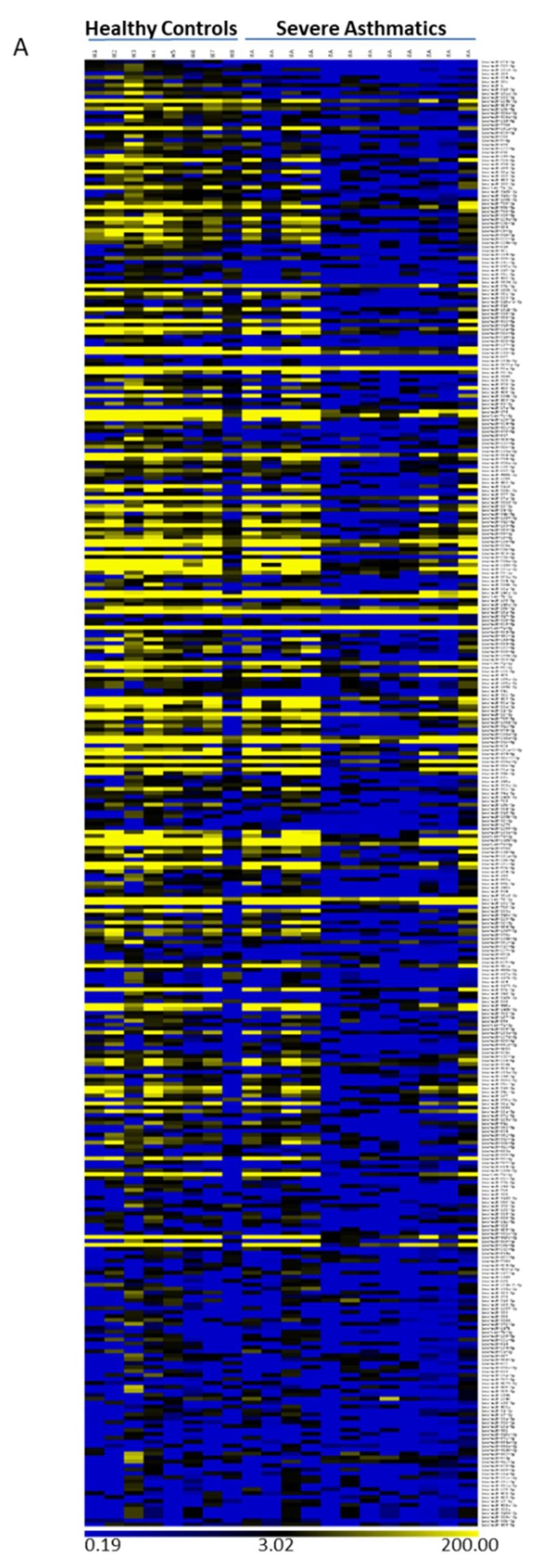

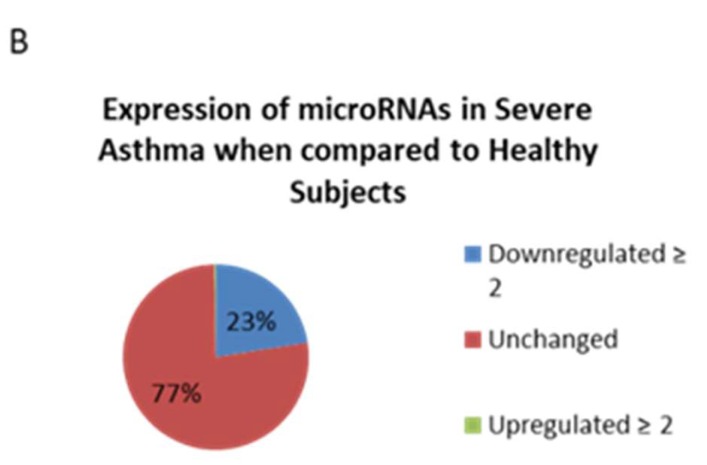
MicroRNA cargo reduced in nanovesicles from severe asthma BALF. (**A**). Heatmap showing unsupervised clustering of SA (*n* = 12) and HC (*n* = 8) (**B**). Comparative levels of microRNAs in nanovesicles according to fold expression (SA/HC). Statistics (*p* value and FDR) according to NIA Array analysis software (*p* ≤ 0.05). SA: Severe asthma. HC: Healthy control. BALF: Bronchoalveolar lavage fluid. FDR: False discovery rate.

RNA sequencing also showed a reduction in the cargo of microRNAs in nanovesicles from SA patients when compared to healthy subjects (Figure 2A).

Statistical analysis of microRNA levels revealed that 90 out of the 373 microRNAs detected by small RNA-sequencing were significantly less present (*p* < 0.05 and FDR ≤ 0.2) in nanovesicles extracted from BALF among SA patients (Supplementary Table S1). Thus, while most microRNA appeared to be unchanged, 23% of microRNA showed a reduction in the load (Figure 2B). Nanovesicles from SA did not show an increase in the loading of any microRNA, which suggests that this observation may be a consequence of a faulty mechanism to specifically load microRNAs in nanovesicles.

### 4.3. Altered microRNA Content of Severe Asthmatic Nanovesicles Affects Cellular Pathways Involved in Airway Inflammation and Remodeling, and Correlates with FEV_1_ and Differential Eosinophil and Neutrophil Infiltration

We then investigated, in silico, the potential pathological effect of the alteration of microRNA nanovesicle cargos on cellular pathways by evaluating the targets predicted to be affected by one or more of the microRNAs most significantly downregulated in severe asthma (*p* ≤ 0.01, FDR ≤ 0.15, ratio SA/HC < 0.33), according to two databases (TargetScan version 6. 2 and mirDB version 4.0) (Supplementary Table S2). Predicted targets were mapped to pathways affecting epithelial cell barrier function and remodeling (focal adhesion, adherence junction, TGF-β signaling, and regulation of the actin cytoskeleton). The decrease in microRNA regulation was predicted to increase genes involved in inflammation and remodeling (i.e., *EGFR*, *NFKB*, *PI3K*, *RAS*, *RAF*, *SOCS1*, *SMADs*, TGF-β receptors, *TNFα,* and *VEGF*. Supplementary Table S2).

**Figure 3:**
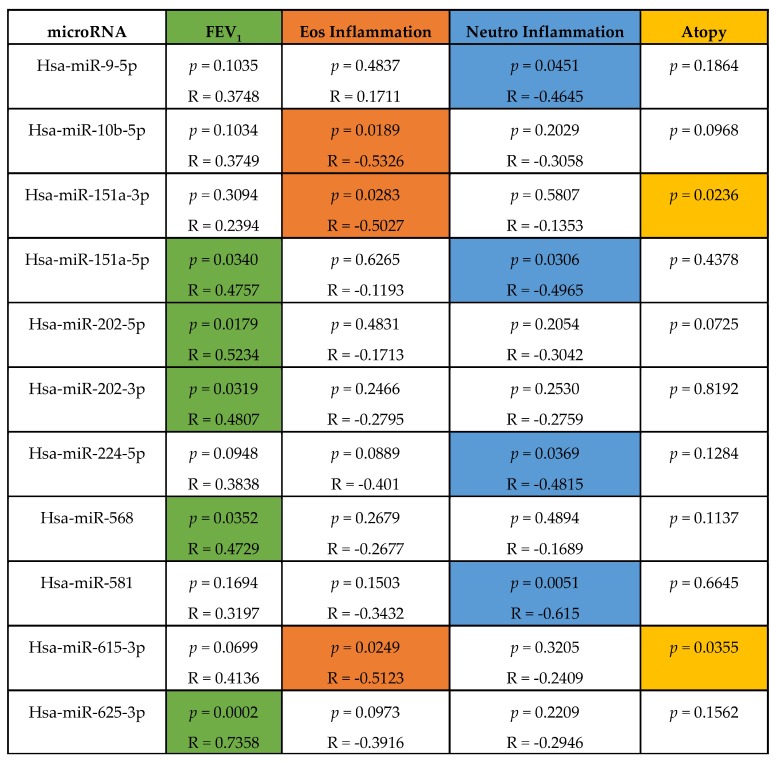
MicroRNAs downregulated in severe asthma correlate to FEV_1_ and immune inflammation. MicroRNAs with SA/HC ratio < 0.33 (*p* ≤ 0.01, FDR ≤ 0.15) in nanovesicles from SA were correlated (Spearman’s rank) with FEV_1_ (green), eosinophilic inflammation (orange), or neutrophilic inflammation (blue)**.** Influence of atopy (yellow) on microRNAs significantly correlated to FEV_1_, eosinophilic inflammation, or neutrophilic inflammation, which were calculated using the Mann-Whitney test (*p* ≤ 0.05).

We, thus, hypothesised that the altered nanovesicle microRNA balance has clinical relevance to severe asthma, as previously shown for lung function (FEV_1_) in mild asthmatic patients [6]. We, therefore, tested whether individual microRNA levels (as RPMs) correlated with FEV_1_ and the BAL percentage differential cell count (Figure 3 and Supplementary Figure S3, *p* ≤ 0.05). Confirming our hypothesis, the reduction in hsa-miR-625-3p, hsa-miR-202-5p, hsa-miR-202-3p, hsa-miR-568, and hsa-miR-151a-5p significantly correlated with a reduction in the predicted FEV_1_ percentage. Moreover, we also found out that hsa-miR-615-3p, hsa-miR-10b-5p and has-miR-151a-3p inversely correlated with the BALF eosinophil percentage and hsa-miR-224-5p, hsa-miR-581, hsa-miR-151a-5p, and hsa-miR-9-5p were also inversely correlated with the neutrophil percentage (Figure 3). For analysis of correlation with neutrophil and eosinophil infiltration, we excluded SA8 and SA1, respectively, since these subjects appear to be outliers, which could skew our analysis.

Furthermore, for those microRNAs that significantly correlated to clinical parameters, we found that has-miR-151a-3p and has-miR-615-3p seem to be differently expressed in atopic subjects compared to non-atopic subjects.

These data suggest that the deficiency of specific microRNAs may have different roles depending on the individual, including altering their lung function or enhancing infiltration of neutrophils in some SA patients or eosinophils in others as well as participating in the heterogenous manifestations of severe asthma pathology.

### 4.4. MicroRNAs Associated with FEV_1_, Neutrophil, and Eosinophil Infiltration Regulate Pathways Relevant to Asthma Pathology

**Figure 4:**
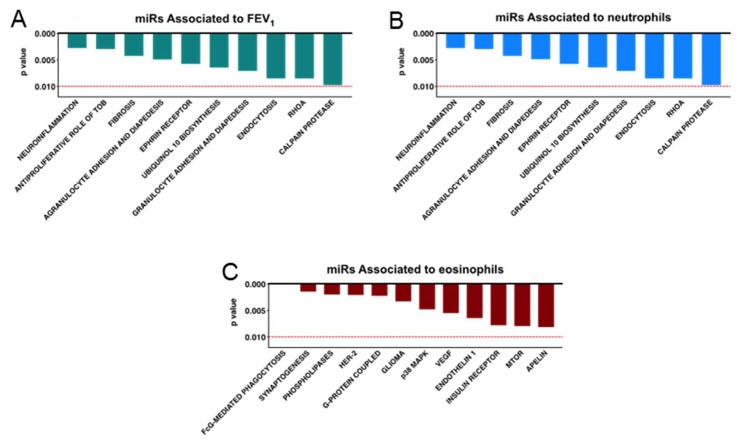
Networks of miRNA target key molecular and cellular pathways related to asthma pathology. Biological pathways were predicted by Ingenuity Pathway Analysis (raw *p* value less than 0.010) to be affected by microRNAs associated with FEV_1_ ((**A**), hsa-mir-151a-5p, hsa-mir-202-3p, hsa-mir-202-5p, hsa-mir-568, hsa-mir-625-3p), neutrophilic inflammation ((**B**), hsa-mir-224-5p, hsa-mir-151a-5p, hsa-mir-581), and eosinophilic inflammation ((**C**), hsa-mir-10b-5p, hsa-mir-151a-3p, and hsa-mir-615-3p).

Next, we determined the pathways associated with each specific network of microRNAs that correlates with lung function as well as neutrophil or eosinophil infiltration to establish whether they affect different cellular mechanisms associated with asthma pathology. We performed Ingenuity Pathway Analysis (IPA) and, interestingly, showed that the pathways linked to both FEV_1_-associated microRNAs and Neutrophilic infiltration are related to the infiltration of immune cells, fibrosis, and virus entry (Figure 4A,B). Our analysis also indicated that microRNAs negatively associated with eosinophil infiltration target candidate mRNAs involved in angiogenesis (VEGF signaling) as well as phagocytosis of macrophages, monocytes, and endothelin 1 (Figure 4C).

Taken together, our data suggest that the alteration of microRNAs in nanovesicles from SA patients affects specific cellular and molecular pathways linked to asthma pathological parameters, such as eosinophilia, neutrophilia, or the reduction of lung function (FEV_1_).

## 5. Discussion

In this study, we looked at the small-RNA cargo of nanovesicles extracted from bronchoalveolar lavage of severe asthmatic patients. We concluded that microRNAs are generally downregulated in severe asthma nanovesicles, potentially due to an aberrant loading mechanism of small RNAs, and this seems to correlate with cellular pathways and clinical features.

Our data suggest that excessive small ribosomal rRNA in nanovesicles may lead to deficient nanovesicle miRNA cargo. This may cause dysregulation of the outlined pathways in SA by worsening lung function. This may be due to up-regulated expression of collagens, TGF-β, and epithelial cell barrier proteins (Smad2/3, cadherins, integrins, and occludins) which, in turn, promote structural airway changes. Impaired epithelial repair is associated with altered activation status and enhances expression of chemokines such as CCL11, CXCL5, and IL-8 [13,14,15], which contribute to recruitment of eosinophils and neutrophils. These effects provide a possible explanation for the correlations observed when correlating the deficiency of different microRNAs with a number of clinical parameters. The microRNAs correlated to FEV_1_ and neutrophil infiltration show some similar targeted pathways that may be involved in asthma pathogenesis, but no overlap with the pathways were affected by microRNAs that correlated with eosinophilic infiltration. This may suggest that FEV_1_ and neutrophilic infiltration could be, from the point of view of the molecular pathology, very different from the eosinophilic infiltration and that microRNAs can help to further stratify patients, together with their clinical parameters. Coherently, microRNAs correlated to eosinophilic infiltration and atopy nearly overlap. The actual biological impact of these microRNAs on their mRNA targets and signaling pathways needs further dissection both mechanistically, in vitro, and functionally, in vivo.

The microRNAs down-regulated in BALF from SA patients are different from the ones dysregulated in mild asthma [6], which suggests that airway intercellular communication and disease mechanisms are quite distinct. Furthermore, there is little overlap with the cellular microRNAs dysregulated in sputum from patients with SA [7], which suggests that the altered microRNA cargo in BALF exosomes is not directly a consequence of lower levels of microRNAs in the inflammatory cells present in the airway lumen and, in addition, highlights the distinction between central and peripheral airway biology in severe asthma [16]. Whether the reduced nanovesicle microRNA content in SA reflects deficient nanovesicle loading or reflects altered activation status of the cellular source, such as alveolar macrophages or bronchial epithelial cells, cannot be determined with the extraction protocol that does not recover the whole nanovesicles but does purify RNA. Future work is needed to relate nanovesicle content to structural airway cell activation and to determine the relationship between nanovesicle microRNA cargo and distinct phenotypes or endotypes of severe asthma [7,8].

We report that SA patients have less microRNAs per total RNA extracted from their airway luminal nanovesicles when compared to healthy volunteers. This may influence intercellular signaling and has potential relevance to disease severity. Our results do not allow us to establish whether poor microRNA quantities are a cause or a consequence of severe asthma features, even though microRNAs in BALF nanovesicles may be a valid biomarker to define biological disease severity and monitor the biological impact of interventional therapies.

## Figures and Tables

**Table 1:** Subject characteristics. F: Female. M: Male. FEV_1_%: forced expiratory volume in 1 s percentage of predicted. Atopy was defined as at least one positive skin prick test response to aeroallergens with testing for *Aspergillus fumigatus, Alternaria taenia,* mixed grass pollen, birch pollen, mixed tree pollen, oil seed rape polen, *Dermatophagoides pteronyssinus, Dermatophagoides farinae*, feathers, and animal allergens (cat, dog, horse, and rabbit). ACQ = Asthma Control Questionnaire. Cell percentages (Neutrophils [Neut] and Eosinophils [Eos]) related to Bronchoalveolar lavage (BAL) differential cell counts.

ID	Sex	Age	Atopy	FEV_1_%	Eos %	Neut %	ACQ	GINA Step
H1	F	51	N	124	0.25	2.5	0	0
H2	M	50	N	94	0	0.5	0	0
H3	F	44	N	88	0	1.8	0	0
H4	F	38	N	97	0.5	3.5	0	0
H5	F	58	N	137	0.25	4.5	0	0
H6	M	29	N	85.3	0.25	4.3	0	0
H7	M	26	N	104	0	1.25	0	0
H8	F	31	Y	110	1	0.75	0	0
SA1	M	62	N	53	9.3	10	4.2	4
SA2	F	31	N	57	0	2.8	4	5
SA3	M	59	N	47.5	0.5	3.3	4.29	5
SA4	F	21	Y	46	0	8.3	3.6	5
SA5	F	63	N	77.8	2	10	3.29	4
SA6	F	43	Y	46.5	0.3	3.3	3.57	4
SA7	F	26	N	71.5	0	4.3	1.71	4
SA8	M	61	Y	88	4.3	28.3	3.3	4
SA9	F	58	N	56	1.25	4.5	2.72	4
SA10	F	45	Y	94	0.25	7.8	2.14	4
SA11	F	37	Y	77	1.5	1	4.2	4
SA12	F	51	Y	77	0	6.5	2.86	4

## Supplementary Materials

The following are available online at https://www.mdpi.com/2311-553X/5/4/51/s1. Figure S1: Characterisation of exosomes, Figure S2: RNA quality control of the samples, Figure S3: Correlations of microRNA and clinical parameters, Table S1: 90 microRNAs are downregulated in severe asthmatic BAL exosomal microRNA, Table S2: Pathways dysregulated in asthma.
