# Supplementary material for: Small RNA Species and microRNA Profiles are Altered in Severe Asthma Nanovesicles from Broncho Alveolar Lavage and Associate with Impaired Lung Function and Inflammation
Source: Noncoding RNA. 2019 Nov 2;5(4):51. doi: 10.3390/ncrna5040051 (PMC6958500; doi:10.3390/ncrna5040051)
Supplement: Supplementary file 1 [file ncrna-05-00051-s001.pdf]

Supplementary Figures and Tables

Supplementary Figure S1

A

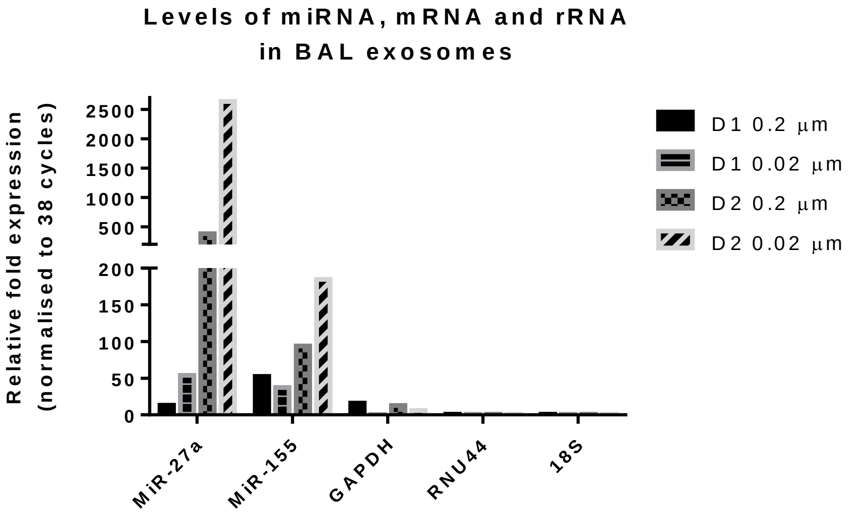

B

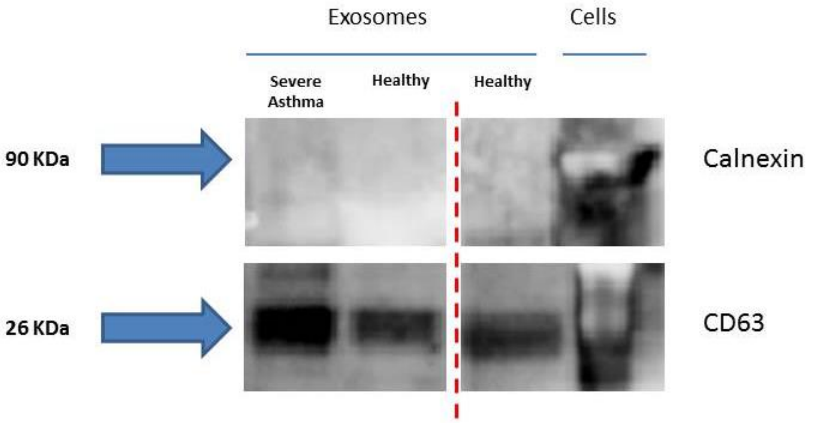

Supplementary Figure S2

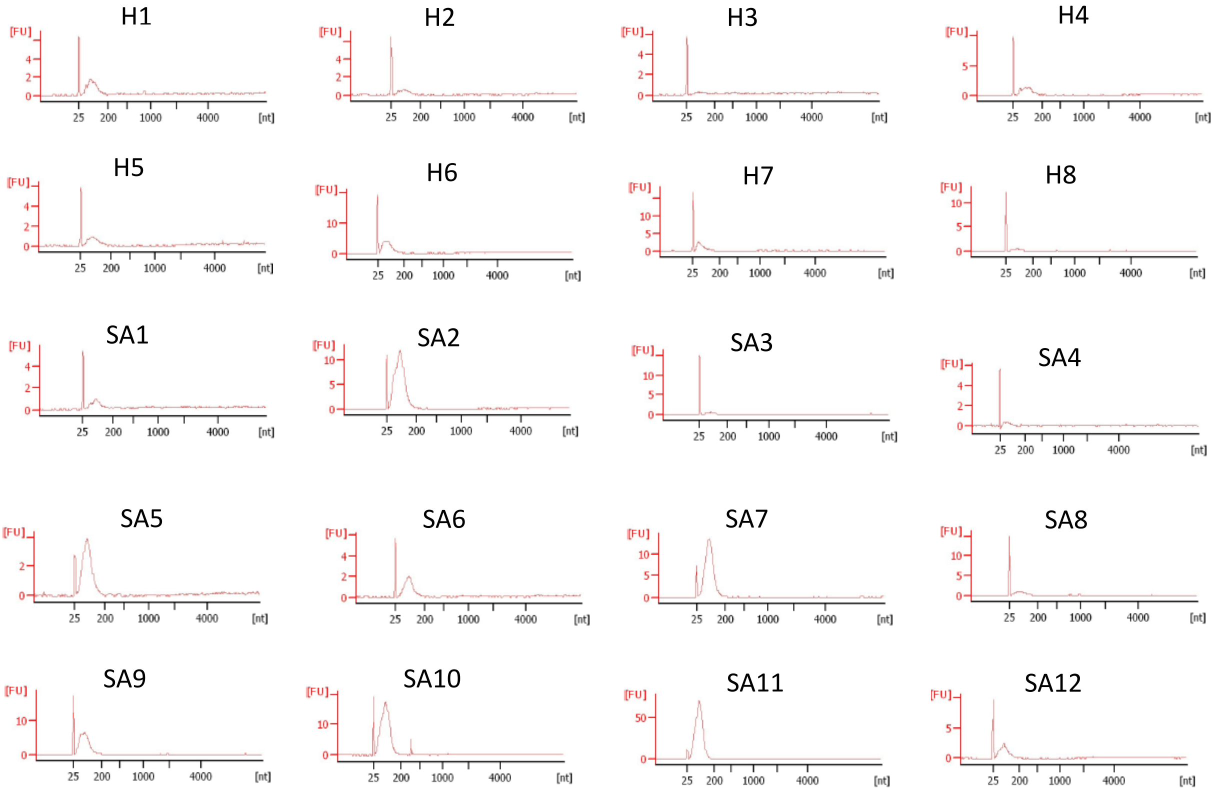

Supplementary Figure S3

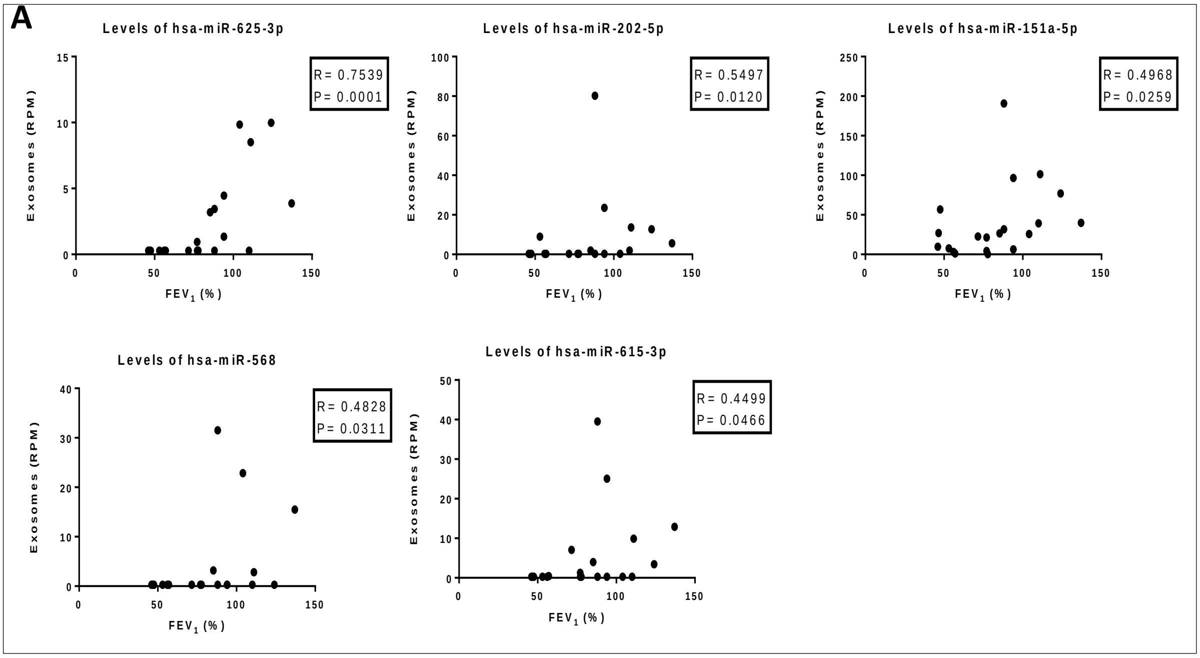

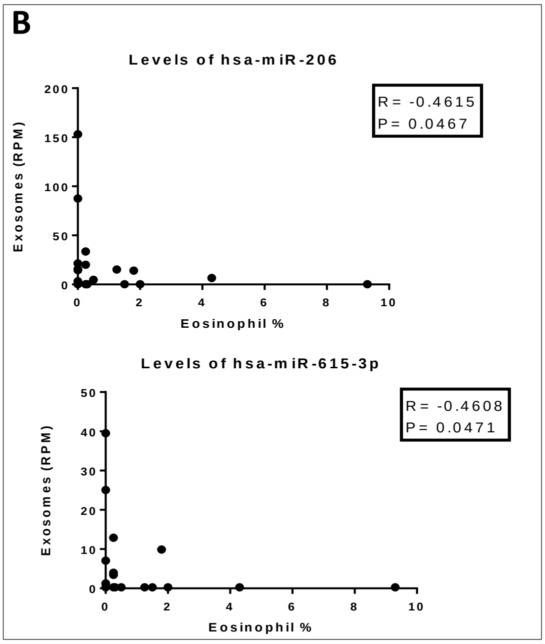

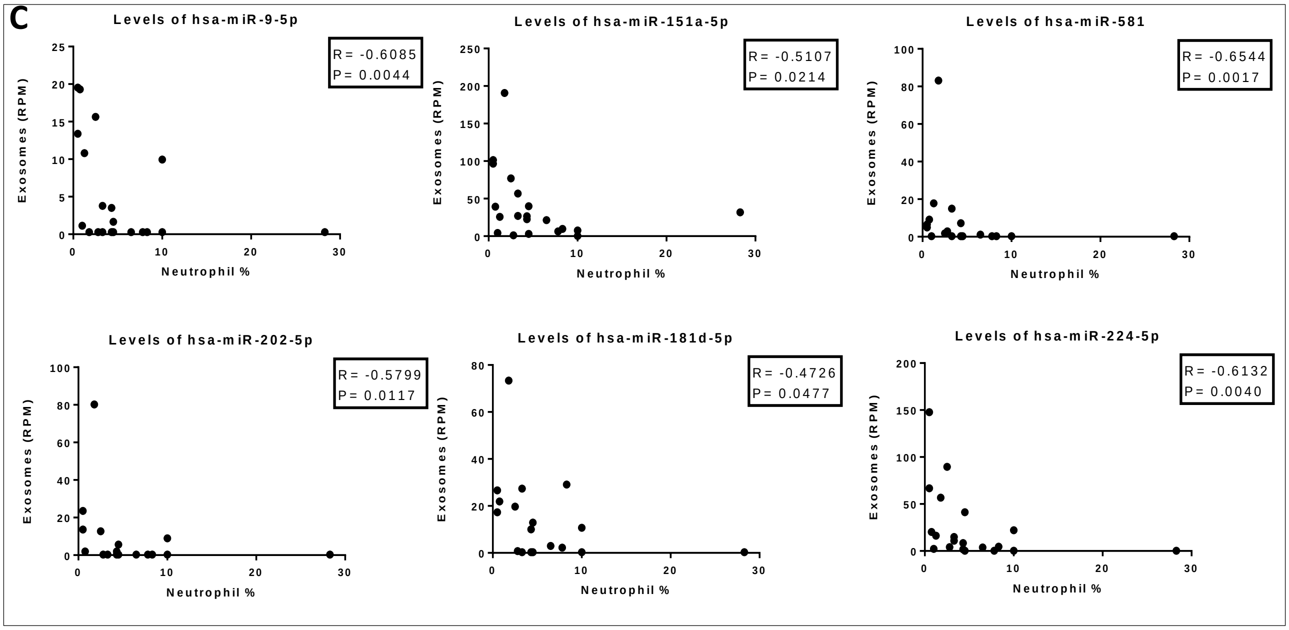

Supplementary Table ST1 – 90 microRNAs are downregulated in severe asthmatic BAL exosomal microRNA

Mature miRNA Name	Anova P-value	Anova FDR	Fold Changes (Healthy vs SA)
hsa-miR-625-3p	0.00004	0.01351	12.73708735
hsa-miR-202-5p	0.00032	0.05978	17.30941106
hsa-miR-181d-5p	0.00097	0.10592	3.90773668
hsa-miR-568	0.00157	0.10592	33.6846983
hsa-miR-224-5p	0.0019	0.10592	9.17330953
hsa-miR-581	0.00196	0.10592	9.113094659
hsa-miR-1	0.00222	0.10592	5.147353113
hsa-miR-615-3p	0.00227	0.10592	12.64794731
hsa-miR-151a-5p	0.00347	0.14016	4.677970418
hsa-miR-202-3p	0.00376	0.14016	15.47127905
hsa-miR-125b-5p	0.00598	0.15237	3.646614539
hsa-miR-425-3p	0.00613	0.15237	2.859058738
hsa-miR-10b-5p	0.00615	0.15237	3.815368896
hsa-miR-500a-5p	0.00724	0.15237	5.848936075
hsa-miR-520a-3p	0.00735	0.15237	9.131554923
hsa-miR-145-5p	0.0074	0.15237	14.14228551
hsa-miR-7706	0.00775	0.15237	4.251400485
hsa-miR-151a-3p	0.00777	0.15237	3.985706942
hsa-miR-628-3p	0.00807	0.15237	4.19099152
hsa-miR-206	0.00817	0.15237	8.075113169
hsa-miR-9-5p	0.0088	0.15287	6.601860461
hsa-miR-578	0.00918	0.15287	4.88633972
hsa-miR-122-5p	0.00943	0.15287	5.650117117
hsa-miR-646	0.00984	0.15295	8.332704341
hsa-miR-149-5p	0.01103	0.1615	3.565116981
hsa-miR-205-5p	0.01126	0.1615	4.494590137
hsa-miR-370-3p	0.01225	0.16518	6.672305042
hsa-miR-598-3p	0.0124	0.16518	4.296117834
hsa-miR-30a-3p	0.01357	0.1684	3.328264378
hsa-miR-552-3p	0.01372	0.1684	6.362617624
hsa-miR-493-3p	0.01541	0.1684	7.516934698
hsa-miR-502-3p	0.0161	0.1684	2.04905949
hsa-let-7e-5p	0.01641	0.1684	3.9632466
hsa-miR-516b-5p	0.01645	0.1684	8.65840658
hsa-miR-518c-5p	0.01656	0.1684	5.358308115
hsa-miR-106b-5p	0.01658	0.1684	3.279617121
hsa-miR-708-3p	0.0167	0.1684	2.284130907
hsa-miR-99b-5p	0.01759	0.17185	3.02832834
hsa-miR-708-5p	0.01797	0.17185	1.583111582
hsa-miR-339-5p	0.01872	0.17188	4.173525589
hsa-miR-125a-5p	0.01917	0.17188	2.848101262
hsa-miR-23b-3p	0.01935	0.17188	2.555333689
hsa-miR-484	0.02087	0.18106	3.094496658
hsa-miR-25-3p	0.02269	0.187	2.463719385
hsa-miR-340-3p	0.02323	0.187	3.808979069
hsa-miR-222-3p	0.02481	0.187	3.523702049
hsa-miR-135b-5p	0.02524	0.187	2.409671607
hsa-miR-610	0.02542	0.187	7.660760307
hsa-miR-421	0.02625	0.187	5.084263677
hsa-miR-154-5p	0.02631	0.187	5.324262368
hsa-miR-339-3p	0.02667	0.187	2.772589834
hsa-miR-141-3p	0.02826	0.187	4.522902385
hsa-miR-642a-3p	0.02872	0.187	3.962289923
hsa-miR-512-3p	0.0296	0.187	5.740633397
hsa-miR-29c-5p	0.02977	0.187	1.861863266
hsa-miR-432-3p	0.0298	0.187	4.747031935
hsa-miR-3934-5p	0.02992	0.187	3.412651069
hsa-miR-27b-3p	0.03009	0.187	3.029741361
hsa-miR-196b-5p	0.03045	0.187	5.258139059
hsa-miR-361-3p	0.03075	0.187	3.19312995
hsa-miR-223-3p	0.03087	0.187	2.002770697
hsa-miR-219a-1-3p	0.03371	0.187	2.487512138
hsa-miR-618	0.03374	0.187	3.005812985
hsa-miR-181b-5p	0.03404	0.187	3.306539992
hsa-miR-330-3p	0.03443	0.187	4.323106426
hsa-miR-590-3p	0.03487	0.187	4.493258792
hsa-miR-532-5p	0.03513	0.187	2.039622436
hsa-miR-345-5p	0.03536	0.187	1.769460107
hsa-miR-10a-5p	0.03561	0.187	3.225234328
hsa-miR-30c-5p	0.03761	0.187	2.82521551
hsa-miR-210-3p	0.03765	0.187	6.707880135
hsa-miR-508-5p	0.03825	0.187	6.434115532
hsa-miR-187-3p	0.03858	0.187	4.374361733
hsa-miR-186-5p	0.03868	0.187	2.934362441
hsa-miR-143-3p	0.03872	0.187	1.749772468
hsa-miR-607	0.03878	0.187	7.362377653
hsa-miR-193b-3p	0.03902	0.187	9.503401498
hsa-miR-3622a-5p	0.0396	0.187	5.058152899
hsa-miR-99a-5p	0.04001	0.187	3.460720561
hsa-miR-28-3p	0.0406	0.187	2.012607232
hsa-miR-3909	0.04153	0.187	5.77088053
hsa-miR-328-3p	0.04208	0.187	1.233765577
hsa-miR-875-3p	0.04209	0.187	5.917943392
hsa-miR-486-5p	0.04331	0.187	2.124673179
hsa-miR-489-3p	0.0435	0.187	5.386910932
hsa-miR-200b-5p	0.0435	0.187	3.416159075
hsa-miR-483-3p	0.04362	0.187	5.053249029
hsa-miR-93-5p	0.04759	0.20172	2.754331525
hsa-miR-27a-5p	0.0494	0.20551	0.155014724
hsa-miR-375	0.04959	0.20551	2.487893854

Supplementary Table ST2: Pathways dysregulated in severe asthma

Adjusted p-value	Pathway	Highlighted targets
0.0059	Chronic myeloid leukemia	PI3K, Sos, Ras, ERK, NFKB, TGF-β, TGFβRI, TGFβRII, Smad3, Smad4, p53
0.0059	Adherens junction	IGF-1R, INSR, MET, Actin, p120ctn, α-Catenin, Rac, TGFβR, ERK, Smad3, Smad4
0.0059	MAPK signalling pathway	SOS, Ras, PKA, MEK1, ERK, NFKB, TNF, IL1, TGFβ, TGFβR, p38, MKP, NLK
0.0059	Focal adhesion	ECM, ITGA, ITGB, Filamin, Paxillin, Actin PI3K, Rac, PAK, Sos, MEK1, ERK1/2
0.0059	Neurotrophin signalling pathway	PI3K, SOS, Ras, Raf, MEKK3, MEK1/2, p38, Erk1/2, NFKB
0.0059	mTOR signalling pathway	INS/IGF, PI3K, mTOR, ERK1/2, VEGF, elF4B, elF4E, REDD1, AMPK, BRAF
0.0101	TGF-beta signalling pathway	TGFβ, TNFα, TGFβRI, TGFβRII, Smad1/5/8, Smad4, Smad 6/7, Smad2/3, ERK, Activin
0.0133	Regulation of actin cytoskeleton	MEK, ERK, Sos, Ras, PI3K, F-Actin, Rac, PAK, MLCK
0.0138	Endocytosis	TGFβ, TGFβR, Smad2/3, RTK, dynamin, clathrin, Smurf2, Smad7, Rab11, rabaptin5, Rab5, Rab22, Alix, VPS25, CHMP1, VPS, Arf
0.0231	Prostate cancer	GF, GFR, PI3K, SOS, Ras, Raf, MEK, ERK, p27, NFKB, mTOR
0.0231	Pancreatic cancer	PI3K, NFKB, Raf, Rac, MEK, ERK, VEGF, p16, Rb, p53, BRCA2, TGFβ, TGFβRI, Smad2/3, Smad4,
0.0231	Melanoma	GF, RTK, Ras, BRAF, Raf, PI3K, MEK, ERK, p53, Rb, E2F
0.0231	Insulin signalling pathway	SOCS, PI3K, GLUT4, PKA, mTOR, SOS, Ras, Raf, MEK1/2, ERK 1/2
0.0266	Glioma	IGF-1, EGFR, PDGFR, Shc, Sos, Ras, PI3K, Raf, MEK, ERK, mTOR, Rb,E2F, p53, PDGF
0.0295	Dilated cardiomyopathy	ITGA, ITGB, Desmin, Titin, SGCD, β1AR, AC, PKA, DHPR, SERCA2a, NCX, TNFα, TGFβ, IGF-1, Lamin, Titn, ACTC1, TPM
